# Dental Management of Self-Injurious Behavior in Lesch–Nyhan Disease: A Patient- and Caregiver-Reported Outcome Study

**DOI:** 10.3390/children13081000

**Published:** 2026-07-29

**Authors:** Claudia Capurro, Stefano Parodi, Simone Buttiglieri, Giulia Romanelli, Caterina Del Buono, Nicola Laffi

**Affiliations:** 1 Pediatric Dentistry and Orthodontics Unit, IRCCS Istituto Giannina Gaslini, 16147 Genoa, Italy; caterinadelbuono@gaslini.org (C.D.B.); nicolalaffi@gaslini.org (N.L.); 2 Epidemiology and Biostatistics Unit, Scientific Directorate, IRCCS Istituto Giannina Gaslini, 16147 Genoa, Italy; stefanoparodi@gaslini.org; 3 Department of Dentistry, Mauriziano Umberto I Hospital, 10128 Turin, Italy; sbuttiglieri@mauriziano.it

**Keywords:** Lesch–Nyhan disease, self-injurious behavior, intraoral devices, dental extraction, quality of life, pediatric dentistry

## Abstract

**Highlights:**

**What are the main findings?**

• This study provides one of the first patient- and caregiver-reported evaluations of dental management for self-injurious behavior in Lesch–Nyhan disease.
• Intraoral protective devices improved perceived reassurance in most families, whereas dental extractions relieved symptoms but did not consistently abolish self-injurious behavior.

**What are the implications of the main findings?**

• Treatment effectiveness should be assessed using both clinical outcomes and patient- and caregiver-reported outcomes.
• These findings support a multidisciplinary, patient-centered approach to dental management while emphasizing the importance of individualized treatment planning.

**Abstract:**

**Background/Objectives**: Self-injurious behavior (SIB) is one of the most disabling manifestations of Lesch–Nyhan disease (LND), frequently involving the oral cavity and severely affecting patients’ quality of life and caregiver burden. Evidence regarding the impact of dental interventions on patient- and caregiver-reported outcomes remains limited. This study investigated the characteristics of SIB in patients with LND and explored the perceived effectiveness, tolerability, and psychosocial impact of intraoral devices and dental extractions. **Methods**: A questionnaire-based observational study was conducted among patients with LND. A purpose-built 32-item questionnaire, developed by dentists with expertise in Special Care Dentistry at Mauriziano Umberto I Hospital (Turin, Italy), was administered to patients attending the Pediatric Dentistry and Orthodontics Unit at IRCCS Istituto Giannina Gaslini (Genoa, Italy). The questionnaire assessed demographic and clinical characteristics, SIB features, dental management strategies, treatment-related complications, and patient- and caregiver-reported outcomes. **Results**: Twenty-four questionnaires were analyzed. SIB was reported in 21 patients (87.5%). Fingers (80%) and lips (70%) were the most frequently affected sites, with multiple anatomical sites involved in 80% of patients. Intraoral devices were used in 14 patients (66.7%), whereas dental extractions were performed in 4 (19%). Among patients treated with intraoral devices, 78.6% reported increased reassurance and protection. Device-related complications occurred in three patients and were mainly periodontal. All patients and caregivers reported relief following dental extraction, although SIB persisted in 50% of cases. **Conclusions**: SIB is highly prevalent among individuals with LND and commonly affects multiple anatomical sites. Intraoral devices are the most frequently adopted treatment strategy and are generally well tolerated, whereas dental extractions were associated with substantial perceived relief but do not necessarily eliminate SIB. Patient- and caregiver-reported outcomes should be considered when evaluating treatment effectiveness.

## 1. Introduction

Lesch–Nyhan disease (LND) is an extremely rare X-linked recessive genetic disorder caused by mutations in the Hypoxanthine Phosphoribosyl transferase 1 gene (*HPRT1*) located on the long arm of the X chromosome (Xq26). This mutation leads to a deficiency of the enzyme hypoxanthine-guanine phosphoribosyl transferase (HGPRT), resulting in impaired purine metabolism and consequent overproduction of uric acid. The reported incidence ranges widely from 1:100,000 to 1:380,000 male live births, with approximately 40 prevalent cases in Italy [1,2].

Clinically, the metabolic disturbance manifests as hyperuricemia, gouty arthritis, and progressive renal impairment. In addition to metabolic abnormalities, LND is characterized by severe neurological dysfunction, including intellectual disability and movement disorders such as dystonia of varying severity, and may also be associated with megaloblastic anemia [1,3,4]. A distinctive behavioral phenotype, with compulsive self-injurious behavior (SIB), is characteristic. This often involves the oral cavity and includes biting of the lips, tongue, fingers, and oral mucosa, despite intact pain perception. These behaviors are frequently exacerbated by psychological stress and, despite patient awareness of their harmful nature, remain largely uncontrollable [5]. Such manifestations significantly impair quality of life and may interfere with feeding and overall health [6,7].

The diagnosis of LND is based on a combination of clinical findings and laboratory investigations, including biochemical evidence of hyperuricemia and hyperuricosuria in association with psychomotor delay. Enzymatic assays, assessing HGPRT activity in erythrocytes or fibroblasts, along with molecular genetic testing, allow for definitive diagnosis. Molecular analysis is particularly valuable for early detection, carrier identification, and prenatal diagnosis, which can be performed through amniocentesis between the 15th and 18th weeks of gestation or chorionic villus sampling between the 10th and 12th weeks [8,9].

Although affected individuals are usually asymptomatic at birth, clinical manifestations emerge during early infancy with variable severity. In milder forms, symptoms may be attenuated or even absent [1].

Neurological manifestations typically appear between 3 and 6 months of age and include psychomotor delay, hypotonia, and the progressive development of severe action dystonia. As patients grow, they exhibit marked impairment in motor development, including difficulty maintaining head control, sitting, standing, or walking. Additional features include involuntary movements such as choreoathetosis and ballism, often accompanied by dysarthria, dysphagia, opisthotonos, and spasticity [7].

SIB is one of the most disabling and pathognomonic manifestations of LND and typically emerges with the eruption of the primary dentition. Although its pathophysiology remains incompletely understood, increasing evidence suggests that the neurological and behavioral phenotype of LND is associated with developmental abnormalities affecting dopaminergic pathways within the basal ganglia. This developmental disruption, rather than a classical neurodegenerative process, may explain the limited efficacy of dopamine replacement therapies [10,11,12].

SIB often requires continuous supervision and leads to repeated hospitalizations and invasive interventions. One of the distinct features of SIB is the presence of pain and the display of remorse after an episode. Patients display an inability to overcome a compulsive desire to hurt themselves, both in the form of physical injury and/or emotional distress. This condition can cause considerable concern among caregivers and may place affected patients at increased risk of complications [9].

Despite its clinical relevance, there is currently no universally accepted standard of care for the management of SIB and therapeutic approaches remain largely empirical [13].

From a dental perspective, the management of SIB in LND patients remains particularly challenging. Therapeutic approaches include pharmacological, orthodontic, and surgical interventions. In severe cases, extraction of primary or permanent teeth may be required to prevent ongoing tissue damage [14]. However, more conservative strategies, such as the use of intraoral protective devices (bite guards), have been proposed to reduce self-mutilation. Despite potential complications, including the risk of fungal infections and the need for rigorous oral hygiene, these devices currently represent one of the most effective preventive options [15].

However, evidence supporting the long-term efficacy and impact of these devices on patients’ quality of life remains limited, with most available data deriving from case reports or small case series. This highlights the need for investigation into conservative, patient-centered approaches that can reduce morbidity while preserving oral function.

In this context, the present study aims to investigate the characteristics of SIB in patients with LND and to explore patient- and caregiver-reported outcomes associated with intraoral protective devices and dental extractions.

## 2. Materials and Methods

### 2.1. Study Design and Setting

This study was designed as an exploratory questionnaire-based observational study. It was conducted between March and December 2025.

### 2.2. Participants

All patients diagnosed with LND who attended the Pediatric Dentistry and Orthodontics Unit of the Istituto di Ricerca e Cura a Carattere Scientifico (IRCCS) Giannina Gaslini during the recruitment period (between March and December 2025) were considered eligible for inclusion in the study and were invited to participate. Consecutive sampling was adopted.

The eligibility criteria included all patients with a confirmed diagnosis of LND, regardless of age. Individuals without an established diagnosis of LND or who declined to provide informed consent were excluded.

Participation in the study was voluntary and written informed consent was obtained from parents or legal guardians prior to enrollment. Age-appropriate assent was also obtained from the patients, whenever possible. In addition, specific written informed consent for the publication of clinical photographs was obtained from the patients’ legal guardians.

### 2.3. Data Collection and Questionnaire Design

Given the absence of disease-specific validated instruments for evaluating dental management of SIB in LND, a purpose-built questionnaire was developed for this study by dentists with expertise in Special Care Dentistry at Mauriziano Umberto I Hospital, Turin, Italy, and subsequently administered to patients and caregivers at the Pediatric Dentistry and Orthodontics Unit of IRCCS Istituto Giannina Gaslini, Genoa, Italy. The questionnaire consisted of 32 items exploring demographic characteristics, clinical history, self-injurious behavior, treatment-related complications, and patient- and caregiver-reported outcomes associated with intraoral devices and dental extractions. Parents or caregivers were asked to complete the questionnaire during scheduled dental visits.

The development of the questionnaires consisted of five stages:

(1) Observation: The behaviors of patients with LND and their caregivers were observed during the dental visits, including interactions in the waiting room, in the treatment area and at discharge.
(2) Identification of key issues: Key issues related to the well-being of patients with LND and their caregivers, particularly in relation to the management of SIB through dental interventions, were identified.
(3) Questionnaire development: The questionnaire items were designed by dentists with expertise in Special Care Dentistry at Mauriziano Umberto I Hospital, Turin, Italy, to collect information on demographic and clinical characteristics, features of SIB, types of dental treatments received, tolerability and management of intraoral devices when applicable, treatment-related complications, the psychosocial impact on patients and caregivers, and the perceived effectiveness of the interventions.
(4) Content validation: The questionnaire was reviewed by two specialists from the Pediatric Dentistry and Orthodontics Unit at IRCCS Istituto Giannina to assess its comprehensiveness, relevance, and clarity.
(5) Revision and finalization: The questionnaire was revised based on the specialists’ feedback and finalized for administration. It was developed and administered in Italian. An English version was subsequently prepared solely for publication and was not used for participant recruitment or data collection. Consequently, a formal forward–backward translation procedure was not performed. The English version of the questionnaire is provided as Supplementary Material S1. The original Italian version is available as Supplementary Material S2.

The questionnaire was specifically developed for this exploratory study following a structured multistep process. Owing to the rarity of LND and the limited number of eligible patients, formal psychometric validation, including pilot testing with patients or caregivers, cognitive debriefing, construct validity assessment, and test–retest reliability, was not feasible. Instead, the questionnaire underwent expert review by dentists experienced in Special Care Dentistry to evaluate its clinical relevance, clarity, comprehensibility, and feasibility. Their feedback was incorporated into the final version.

### 2.4. Outcome Measures

The primary outcomes were to investigate the presence of SIB among patients with LND, to identify the most common sites of self-injury, to describe the main treatment strategies adopted to manage the consequences of SIB, and to assess the impact of these interventions on the physical and psychological well-being of LND patients and their caregivers.

Secondary outcomes included treatment tolerability, the occurrence of adverse events, and patient- and caregiver- reported satisfaction regarding the interventions.

### 2.5. Data Analysis

All data were collected and processed in accordance with the European General Data Protection Regulation (EU Regulation 2016/679). Responses were anonymized and analyzed in aggregated form to ensure participant confidentiality.

Qualitative variables were described as absolute and relative frequencies, while quantitative ones were described as means and standard deviation or median and interquartile range (IQR) in the presence of non-Gaussian distributions. Normality was assessed by the visual inspection of the corresponding histograms. Missing data were not imputed, and descriptive analyses were performed using the available observations for each variable. Percentages were calculated using the number of valid responses as the denominator for each questionnaire item. Owing to the limited sample size and the exploratory nature of the study, no inferential statistical comparisons were planned or performed.

All statistical analyses were carried out by STATA for Windows statistical package (version MP 18.0, Stata Corp LLC, College Station, TX, USA).

## 3. Results

**Figure 1:**
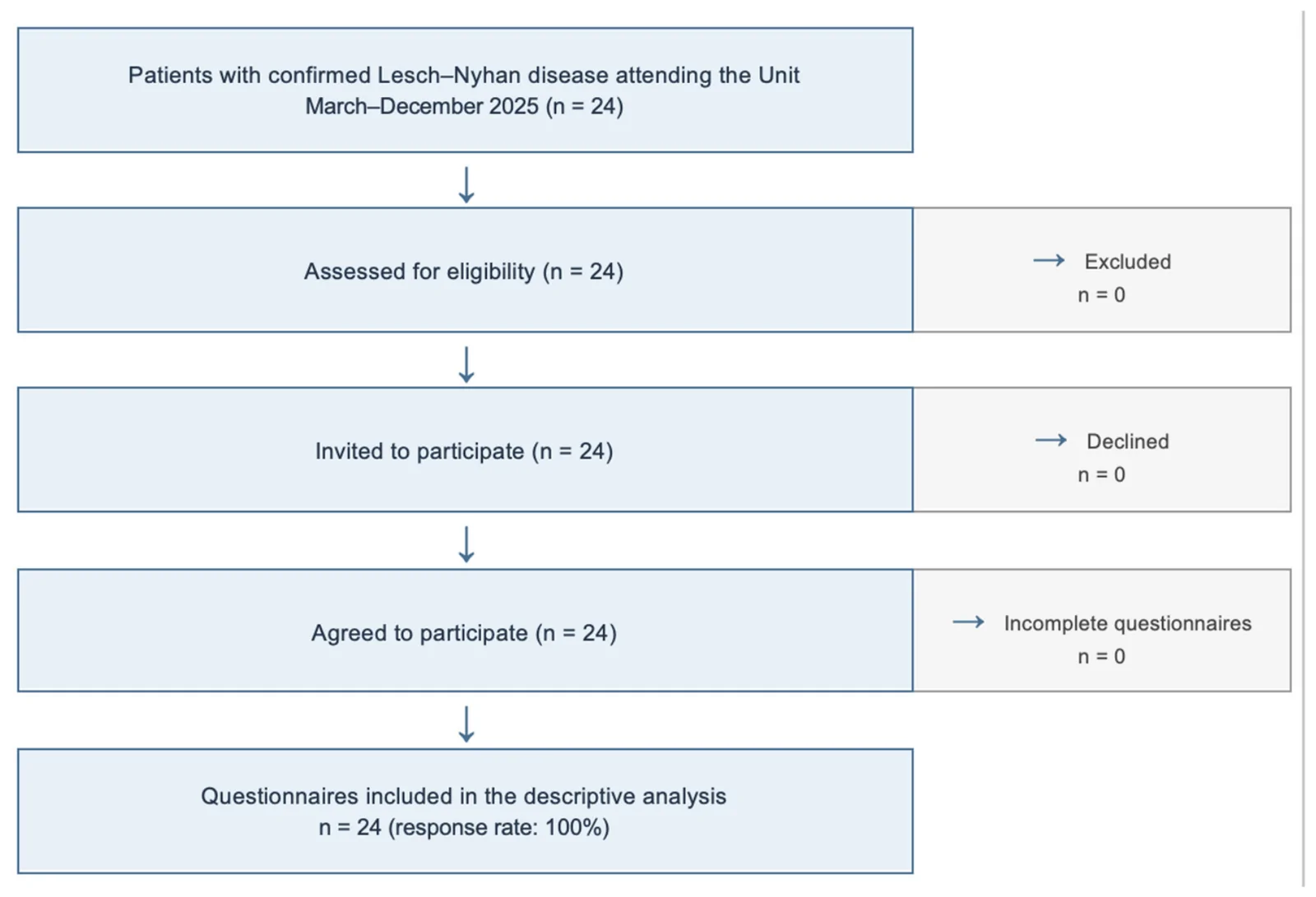
Flow diagram of participant recruitment and inclusion in the study.

All eligible patients agreed to participate and completed the questionnaire, resulting in an overall response rate of 100%. Figure 1 summarizes the participant recruitment process, including eligibility assessment, study inclusion, and questionnaire completion.

Data from a total of 24 completed questionnaires were analyzed. Most questionnaire responses were recorded as yes/no binary variables (items: 6, 9, 12–21, 23–32, Supplementary Material S1), although categorical polytomous variables (item 5 and 8) and quantitative variables (items 3, 4, 7 and 12) were also included when appropriate.

The mean age of the participants at the time of questionnaire completion was 21.1 years (range: 2–51 years).

**Figure 2:**
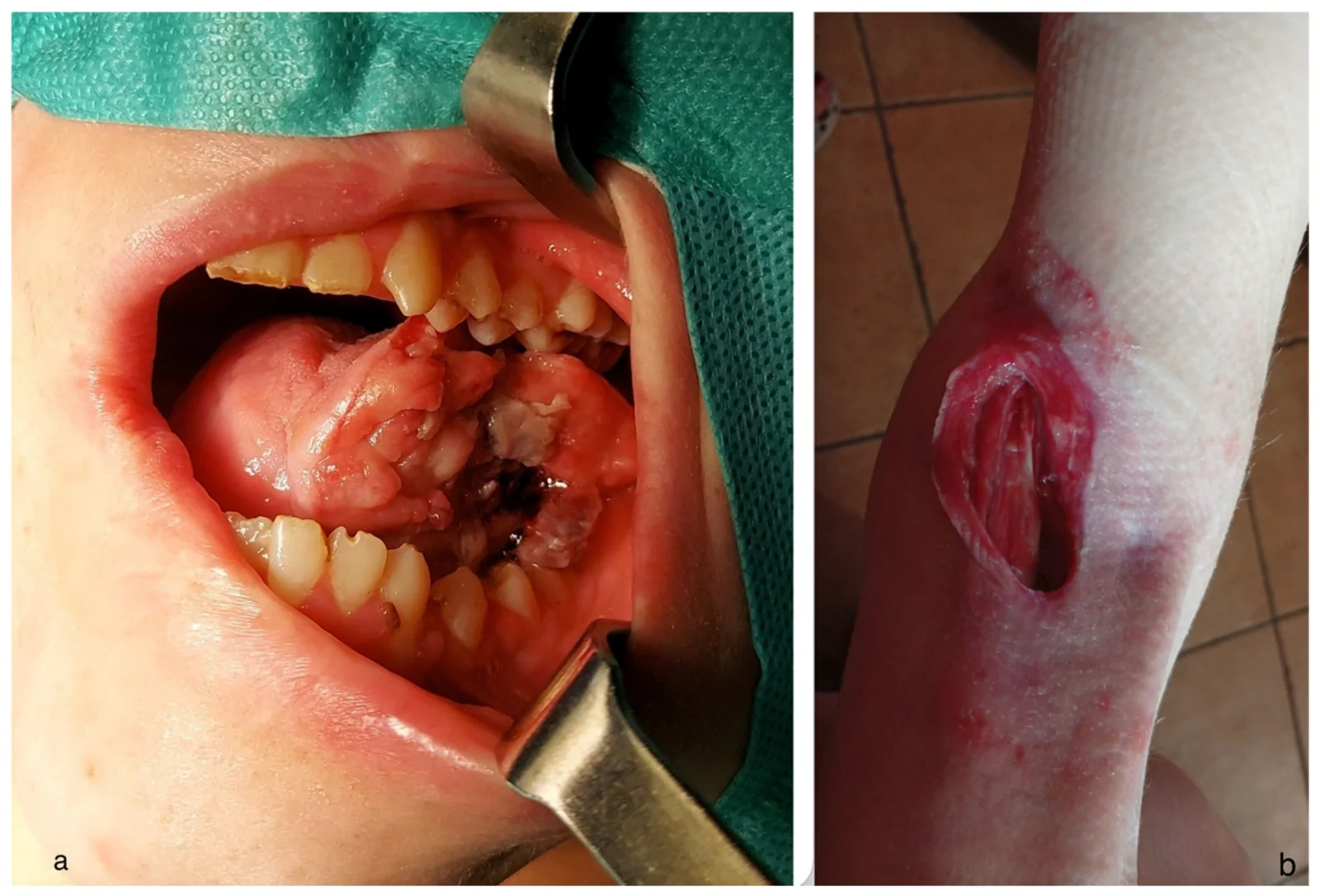
Representative manifestations of self-injurious behavior in patients with Lesch–Nyhan disease. (**a**) Extensive self-inflicted traumatic injury of the tongue. (**b**) Severe self-inflicted soft tissue injury involving the upper limb.

Characteristic symptoms of the disease were reported to appear before 1 year of age in 62.5% of subjects, with a mean age at symptom onset of 26 months (range: 2–168 months; median: 6 months). SIB was reported in 21 of the 24 patients (87.5%). Data on age at SIB onset were available for 20 patients. SIB developed before 1 year of age in 1 patient (5%), between 1 and 16 years of age in 16 patients (80%), and after 16 years of age in 3 patients (15%), at 17, 20, and 30 years of age, respectively. The mean age at SIB onset was 6.4 years. Representative examples of oral and extraoral manifestations of SIB in patients with LND are shown in Figure 2.

The diagnosis was confirmed within the first year of life in 25% of cases, with a mean age at diagnosis of 6.4 years. In only two cases the diagnosis was established before the onset of symptoms.

SIB was observed in 21 of the 24 subjects included in the analysis (87.5%). Information regarding the anatomical location of self-inflicted lesions was available for 20 subjects. The fingers were the most frequently affected site (80%), followed by the lips (70%), tongue (35%), and other anatomical sites (40%), as shown in Table 1.

Simultaneous involvement of multiple anatomical sites was observed in 80% of patients (16/20). Specifically, 8 patients (40%) presented lesions at two anatomical sites, 7 (35%) at three sites, and 1 (5%) at all investigated sites. Only 4 patients (20%) reported self-injury involving a single anatomical site.

**Figure 3:**
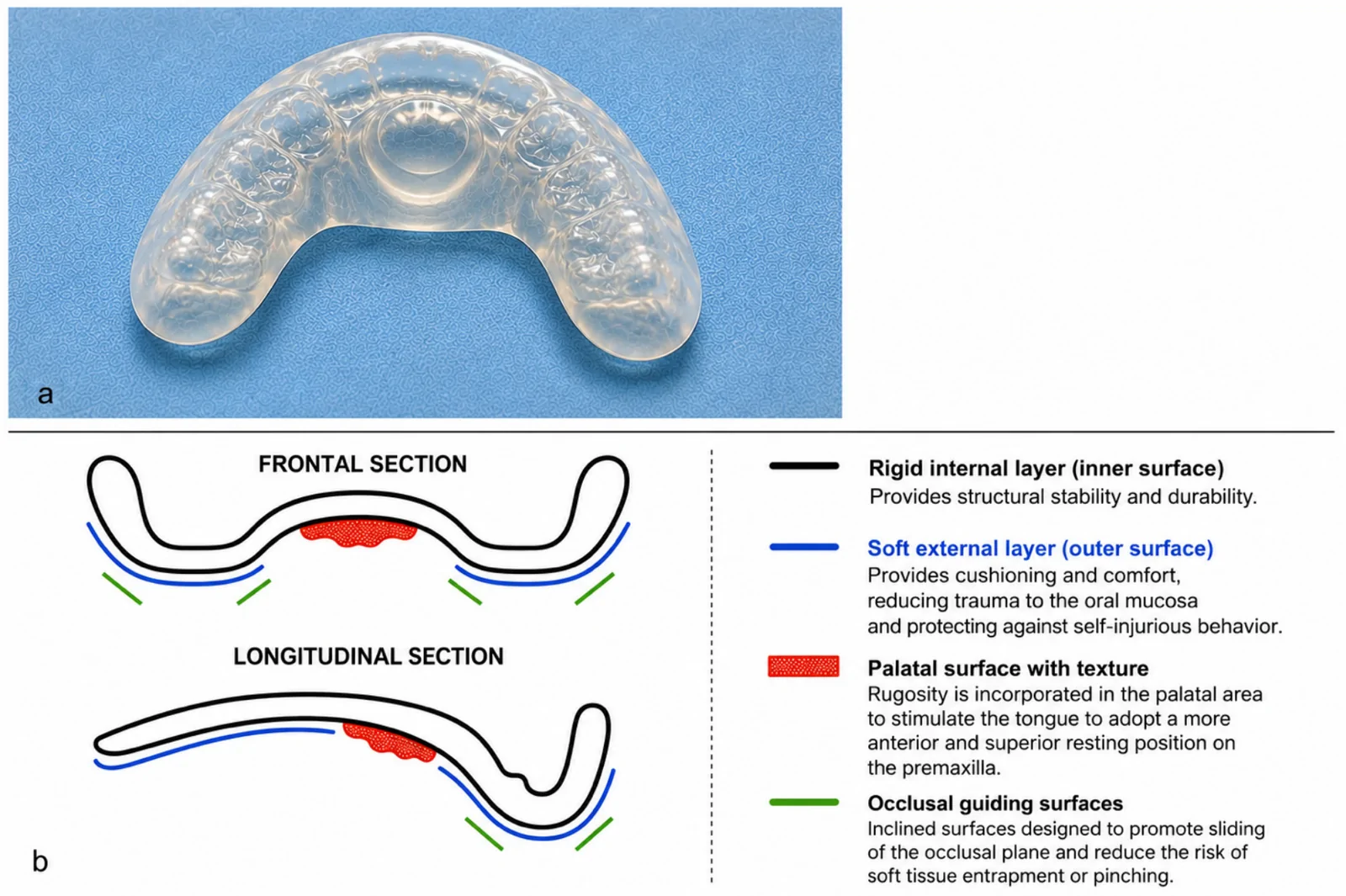
Intraoral protective device used for the management of self-injurious behavior in patients with Lesch–Nyhan disease. (**a**) Occlusal view of the intraoral protective device. (**b**) Schematic representation of the main design features of the device.

The use of a protective intraoral device (bite guard) was reported in 14 of the 21 subjects with SIB (66.7%). Figure 3 illustrates the intraoral protective device most used in our clinical practice and its main design characteristics.

Dental extractions were reported in 4 subjects. Of these, 3 patients underwent both dental extractions and treatment with an intraoral device, while 1 patient was treated with dental extractions alone.

Six of the 21 subjects reporting SIB had not undergone either of the two treatment approaches.

Among patients treated with an intraoral device, the mean age at treatment initiation was 10.5 years (range: 2–26 years). The number of devices used up to the time of questionnaire completion ranged from 1 to 20.

Among the 14 patients treated with an intraoral device, aesthetic or social discomfort was reported by only 2 patients and 2 caregivers (14.3%). Difficulties in managing the device were reported by 5 families (35.7%). Treatment-related complications were reported in 3 patients. Overall, a total of five complications were documented: all three patients reported negative periodontal effects, one reported temporomandibular joint symptoms, and one reported gastrointestinal complications.

Overall, 78.6% of patients and caregivers reported feeling calmer, more reassured, or better protected because of using the intraoral device.

The age at the time of dental extraction varied considerably, ranging from 2.5 to 30 years (2.5, 8, 22, and 30 years, respectively). In all cases, the procedure was completed in a single session. Prior to treatment, all families (100%) reported concerns or fears related to the intervention, whereas such concerns were reported by only one patient.

Following dental extraction, all patients and caregivers reported a sense of relief. However, SIB persisted in 2 of the 4 patients who underwent extraction (50%).

Among patients treated with dental extraction, tongue involvement was present in all cases (4/4, 100%), while lip and finger involvement were observed in 3 of the 4 patients (75%). Three patients (75%) presented lesions affecting at least three anatomical sites, whereas the remaining patient exhibited lesions at two sites.

No caregivers reported aesthetic or social concerns following dental extraction, whereas such concerns were reported by one patient (25%). No temporomandibular joint symptoms were reported, and only one patient reported gastrointestinal symptoms. A summary of patient- and caregiver-reported outcomes associated with intraoral protective devices and dental extractions is presented in Table 2.

## 4. Discussion

LND is one of the rare neurological disorders most strongly associated with SIB, a hallmark manifestation that substantially affects oral health, quality of life, and caregiver burden. Previous studies have reported SIB in up to 85–90% of affected individuals, making it one of the most distinctive clinical features of the disease and a major source of morbidity and family distress [5,16]. In the present study, SIB was reported in 87.5% of participants, confirming its high prevalence and clinical relevance in our cohort.

Although the absolute number of participants included in this study may appear limited, the findings should be interpreted in the context of the extreme rarity of the disease. To date, no mandatory national registry for LND exists in Italy, and available estimates suggest a very small affected population. The present cohort included 24 patients, corresponding to more than half of the estimated Italian population affected by LND (approximately 40 patients). While this represents a major strength of the study given the rarity of the condition, participants were recruited from a single national referral center, and the findings should therefore be interpreted with appropriate caution. The limited sample size remains an unavoidable methodological limitation but also reflects the intrinsic difficulties associated with conducting research on ultra-rare disorders.

The mean age at onset of SIB observed in the present study is consistent with previous reports. SIB typically emerges during early childhood, frequently in association with the eruption of the primary dentition, and generally increases in severity throughout childhood and adolescence [15,17,18]. It is plausible that the presence of functional teeth facilitates the development of the most severe forms of oral self-injury. Furthermore, progressive maturation of motor abilities may contribute to the development of increasingly structured and repetitive self-harming behaviors.

A particularly noteworthy finding concerns the anatomical distribution of lesions. Most patients exhibited simultaneous involvement of multiple anatomical sites, whereas only a minority presented injuries confined to a single area. Similar patterns have been reported in previous studies describing lesions involving the lips, tongue, fingers, oral mucosa, and other body regions [5,7]. These findings support the concept that SIB in LND should not be interpreted exclusively as a dental problem but rather as a complex neurobehavioral manifestation affecting multiple body sites. This observation is particularly relevant when interpreting the outcomes of irreversible interventions such as dental extraction.

Protective intraoral devices represented the most frequently adopted therapeutic approach in our population. The lower mean age at initiation of bite-guard therapy compared with dental extraction suggests that intraoral devices are generally considered first-line treatment. This finding appears consistent with current clinical recommendations, which favor conservative and reversible interventions whenever possible [6,15,19,20]. In pediatric patients, preservation of the dentition remains particularly important for craniofacial development, mastication, speech, and long-term oral health, further supporting the preference for non-invasive approaches.

Interestingly, three of the four patients who underwent dental extraction had previously been treated with an intraoral device. This finding suggests the existence of a progressive therapeutic pathway in which irreversible procedures are considered only when conservative approaches fail to adequately control tissue damage. Similar treatment trends have been described in the literature, where dental extraction is generally reserved for severe, refractory, or life-threatening cases of oral self-injury [19,21,22].

Both intraoral devices and dental extractions were associated with a substantial absence of major complications. However, their perceived impact differed considerably. More than three-quarters of patients and caregivers reported feeling safer and more reassured during the use of an intraoral device, whereas all patients and caregivers reported a sense of relief following dental extraction. This finding highlights the importance of evaluating treatment success not only through clinical outcomes but also through patient- and caregiver-reported outcomes.

Several factors may explain the greater reassurance associated with extraction. Intraoral devices require daily cooperation, regular maintenance, and periodic replacement, and they may be lost, damaged, or temporarily removed. Moreover, despite reducing tissue injury, they do not eliminate the possibility of self-harm episodes. Consequently, families may continue to experience anxiety regarding the effectiveness of the device. In contrast, dental extraction immediately removes the primary traumatic instrument responsible for oral injuries. The relief reported after extraction may reflect not only the clinical efficacy of the procedure but also a reduction in caregiver vigilance burden and fear of future injury. Similar observations have been described in previous reports of severe oral self-mutilation managed by extraction [21,22].

An important finding of the present study is that removal of the dentition through extractions did not necessarily result in resolution of SIB. Indeed, SIB persisted in 50% of patients who underwent extraction. This observation supports the widely accepted hypothesis that self-injury in LND is primarily driven by neurological and behavioral mechanisms rather than by the mere presence of teeth [16,23]. Current evidence suggests that abnormalities involving basal ganglia circuitry and dopaminergic signaling contribute to the persistence of self-injurious behavior, even when the immediate source of oral trauma is removed [24]. Nevertheless, because the questionnaire assessed the persistence of SIB as a whole rather than exclusively oral self-biting, caregivers may also have reported self-injurious behaviors affecting extraoral sites. Therefore, these findings should be interpreted as reflecting the persistence of the overall self-injurious phenotype rather than oral self-injury alone.

Another noteworthy observation concerns patients who exhibited SIB but received neither intraoral devices nor dental extractions. Several explanations may account for this finding. In some cases, the behavior may have been considered clinically mild and therefore not severe enough to warrant intervention. In others, organizational barriers, limited access to specialized dental care, or family preference for non-invasive approaches may have influenced treatment decisions. Regardless of the underlying reason, this finding highlights the heterogeneity of current management pathways and further underscores the lack of standardized protocols for the dental management of LND.

Our findings should also be interpreted in the context of the cross-sectional study by Isola et al. (2022) [5], which investigated the prevalence of oral SIB and the therapeutic approaches adopted in Italian patients with LND. Consistent with their results, dental extraction was frequently adopted as a management strategy for severe oral SIB despite its invasive nature and potential functional consequences. However, whereas Isola et al. primarily described the prevalence of oral SIB and the frequency of the different treatment modalities, the present study complements these observations by specifically evaluating patient- and caregiver-reported outcomes, including perceived treatment effectiveness, quality of life, and satisfaction with different dental management strategies. To the best of our knowledge, the two cohorts are independent, as the studies were conducted in different clinical settings and several years apart.

The findings of this study should be interpreted considering the following limitations. The sample size was necessarily small because of the rarity of the disease. Although the questionnaire underwent expert review to ensure clinical relevance and comprehensibility, it did not undergo formal psychometric validation, including pilot testing, construct validity assessment, or test–retest reliability evaluation. Therefore, it should be considered an exploratory instrument, and future multicenter studies should aim to formally validate it. The retrospective nature of the questionnaire may also have introduced recall bias, as several items relied on patients’ or caregivers’ recollection of events that had occurred years earlier, such as the onset of disease manifestations, self-injurious behavior, and previous treatments. Although caregivers of patients with rare chronic diseases are generally highly involved in long-term care, inaccuracies in recall cannot be completely excluded. Furthermore, the questionnaire did not specifically investigate the persistence of SIB following the introduction of intraoral devices. Consequently, although information regarding perceived effectiveness and treatment-related outcomes was collected, it was not possible to directly compare the persistence of SIB between patients treated with intraoral devices and those who underwent dental extraction. Finally, the limited size of the treatment subgroups precluded robust statistical comparisons and multivariable analyses. Selection bias of the sample should also be considered. Participants were consecutively recruited from a single national referral center, and therefore the study population may not fully represent all individuals with LND in Italy. Although approximately 40 patients are estimated to be affected nationwide, our cohort consisted of patients attending the IRCCS Istituto Giannina Gaslini during the recruitment period who agreed to participate. Consequently, the findings should be interpreted with caution and may not be generalizable to all patients with LND or to healthcare settings with different clinical practices. Moreover, potential selection bias related to treatment allocation should be considered. In routine clinical practice, dental extractions are generally reserved for patients with more severe or refractory SIB after failure of conservative approaches. Therefore, patients treated with dental extraction may have differed substantially from those managed with intraoral devices, limiting direct comparisons between treatment strategies and the interpretation of perceived treatment effectiveness. These findings describe patient- and caregiver-reported outcomes associated with each management strategy and should not be interpreted as evidence of comparative treatment effectiveness.

Therefore, the findings should be regarded as primarily descriptive and exploratory. Nevertheless, the study provides original data on a population rarely investigated in the dental literature and addresses a largely unexplored aspect of care, namely the perceived impact of dental interventions on the well-being of both patients and caregivers.

To the best of our knowledge, this is among the first studies specifically investigating patient- and caregiver-reported outcomes associated with different dental management strategies for SIB in LND.

## 5. Conclusions

This exploratory study provides novel insights into patient- and caregiver-reported outcomes associated with dental management strategies for self-injurious behavior in patients with LND. Intraoral protective devices represented the most commonly adopted conservative approach in our cohort, whereas dental extractions were performed in selected patients with more severe SIB. Because treatment allocation was based on clinical indications rather than randomization, these findings should not be interpreted as evidence of comparative treatment effectiveness.

The persistence of SIB in some patients following dental extraction suggests that dental interventions primarily address the consequences of self-injury rather than the underlying neurobehavioral mechanisms of the disease.

Overall, our findings highlight the value of incorporating patient- and caregiver-reported outcomes alongside traditional clinical measures when evaluating dental management strategies for patients with LND. Further multicenter studies using validated assessment instruments are warranted to confirm and extend these findings. A multidisciplinary approach involving dentists, neurologists, psychologists, rehabilitation specialists, and caregivers remains essential for the long-term management of self-injurious behavior in this patient population.

## Figures and Tables

**Table 1:** Prevalence and anatomical distribution of self-injurious behavior (SIB).

Variable	*n*	%
**Study population**		
Total participants	24	100%
Participants with SIB	21	87.5%
**Characteristics of SIB**		
Participants with available data on SIB location	20	95.2% ^§^
Single anatomical site involved	4	20% *
≥2 anatomical sites involved	16	80% *
Finger involvement	16	80% *
Lip involvement	14	70% *
Tongue involvement	7	35% *
Other sites involved	8	40% *

^§^ Percentage was calculated using the 21 participants with SIB as denominator. * Percentages were calculated using the 20 participants with available data on the anatomical location of SIB as the denominator.

**Table 2:** Patient- and caregiver-reported outcomes associated with intraoral protective devices and dental extractions in patients with Lesch–Nyhan disease.

Variable	Intraoral Device (*n* = 14)	Dental Extraction (*n* = 4)
Mean age at treatment (years)	10.5	15.6
Aesthetic/social concerns reported by patients	2 (14.3%)	1 (25%)
Aesthetic/social concerns reported by caregivers	2 (14.3%)	0 (0%)
Difficulties in treatment management	5 (35.7%)	N/A
Patients with treatment-related complications	3 (21.4%)	1 (25%)
Temporomandibular symptoms	1 (7.1%)	0 (0%)
Gastrointestinal symptoms	1 (7.1%)	1 (25%)
Patients/caregivers feeling reassured or relieved	11 (78.6%)	4 (100%)
Persistence of SIB * after treatment	Not specifically investigated.	2 (50%)

* SIB: self-injurious behavior.

## Data Availability

The datasets generated and/or analyzed during the current study are not publicly available as they are being utilized for ongoing purposes; however, they can be made available by the corresponding authors upon reasonable request.

## Supplementary Materials

The following supporting information can be downloaded at https://www.mdpi.com/article/10.3390/children13081000/s1. Supplementary file S1: Questionnaire—English version; Supplementary file S2: Questionnaire—Original Italian version.

## Abbreviations

The following abbreviations are used in this manuscript:

HGPRT	Hypoxanthine-Guanine Phosphoribosyltransferase
HPRT1	Hypoxanthine Phosphoribosyltransferase 1
IRCCS	Istituto di Ricerca e Cura a Carattere Scientifico
IQR	Interquartile Range
LND	Lesch–Nyhan Disease
SIB	Self-Injurious Behavior
