# Supplementary material for: Dental Management of Self-Injurious Behavior in Lesch–Nyhan Disease: A Patient- and Caregiver-Reported Outcome Study
Source: Children (Basel). 2026 Jul 29;13(8):1000. doi: 10.3390/children13081000 (PMC13511571; doi:10.3390/children13081000)
Supplement: Supplementary file 1 [file children-13-01000-s001.zip › Supplementary Material S1.pdf]

Supplementary Material S1

English Version of the Questionnaire

Questionnaire on Dental Treatments for Self-Injurious Behavior (SIB) in Lesch–Nyhan Disease (LND)

Dear Family,

We kindly ask you to complete this questionnaire regarding your child's condition following dental treatments received to manage the consequences of self-injurious behavior (SIB). The information collected will help us identify the most appropriate clinical approaches for your child and for future patients with Lesch–Nyhan disease.

Date of completion: __________________

Patient Information

1. Patient name or code: __________________

2. Patient date of birth: __________________

3. Age at onset of LND symptoms (months): __________________

4. Age at diagnosis of LND (years): __________________

5. Number of specialists consulted before obtaining the diagnosis of LND:

□ 1–2

□ 3–5

□ 5–10

□ >10

Self-Injurious Behavior (SIB)

6. Presence of self-injurious behavior (SIB):

□ Yes

□ No (if No, end of questionnaire)

7. Age at onset of SIB (years): __________________

8. Anatomical sites affected by self-injury (multiple answers possible):

□ Lips

□ Tongue

□ Fingers

□ Other: __________________

Intraoral Protective Devices

9. Has an intraoral protective device been used?

□ Yes

□ No (if No, proceed to Question 21)

10. Age at initiation of intraoral device therapy (years): __________________

11. Number of intraoral devices used: __________________

12. Were different types of intraoral devices used?

□ Yes

□ No

13. Did the family experience aesthetic or social concerns related to the intraoral device?

□ Yes

□ No

14. Did the patient experience aesthetic or social concerns related to the intraoral device?

□ Yes

□ No

15. Were there any difficulties in managing the intraoral device (e.g., insertion, cleaning, or tolerance)?

□ Yes

□ No

16. Did any temporomandibular joint-related symptoms occur because of the intraoral device (e.g., headache, facial pain, joint noises)?

□ Yes

□ No

17. Did any periodontal problems occur because of the intraoral device (e.g., gingival pain or excessive gingival bleeding)?

□ Yes

□ No

18. Did any gastrointestinal symptoms occur because of the intraoral device (e.g., abdominal pain, indigestion, or reflux)?

□ Yes

□ No

19. Did the patient feel calmer, reassured, or protected while using the intraoral device?

□ Yes

□ No

20. Did the family feel more reassured when the patient was using the intraoral device?

□ Yes

□ No

Dental Extractions

21. Were the teeth responsible for traumatic injuries extracted?

□ Yes

□ No (if No, end of questionnaire)

22. Age at dental extraction (years): __________________

23. Were all dental extractions performed during a single treatment session?

□ Yes

□ No

24. Did the family experience concern or fear before the dental extractions?

□ Yes

□ No

25. Did the patient experience concern or fear before the dental extractions?

□ Yes

□ No

26. Did the family experience aesthetic or social concerns following the dental extractions?

□ Yes

□ No

27. Did the patient experience aesthetic or social concerns following the dental extractions?

□ Yes

□ No

28. Did any temporomandibular joint-related symptoms occur following the dental extractions (e.g., headache, facial pain, or joint noises)?

□ Yes

□ No

29. Did any gastrointestinal symptoms occur following the dental extractions (e.g., abdominal pain, indigestion, or reflux)?

□ Yes

□ No

30. Did the family feel relieved after the dental extractions?

□ Yes

□ No

31. Did the patient feel relieved after the dental extractions?

□ Yes

□ No

32. Did self-injurious behavior (SIB) persist after the dental extractions?

□ Yes

□ No
