# Supplementary material for: Dental Management of Self-Injurious Behavior in Lesch–Nyhan Disease: A Patient- and Caregiver-Reported Outcome Study
Source: Children (Basel). 2026 Jul 29;13(8):1000. doi: 10.3390/children13081000 (PMC13511571; doi:10.3390/children13081000)
Supplement: Supplementary file 1 [file children-13-01000-s001.zip › Supplementary Material S2.pdf]

Supplementary Material S2

QUESTIONARIO SUI TRATTAMENTI ODONTOIATRICI LEGATI ALLA

SINDROME DI LESCH NYHAN (LND)

Rivolto ai familiari dei pazienti

Gentile famiglia, siamo a sottoporvi questo breve questionario anamnestico riguardante le condizioni di vostro figlio a seguito dei trattamenti odontoiatrici cui è stato sottoposto per contrastare gli esiti dei comportamenti autolesionistici (SIB), al fine di valutare il miglior approccio clinico da mettere in atto in futuro, per lui e per tutti i pazienti che verranno.

Data Compilazione: __________________

1. Cognome Nome Paziente:

2. Data di nascita del Paziente:

3. Età dell'esordio dei sintomi della malattia LND (in mesi):

4. Età diagnosi LND (in anni):

5. Numero specialisti necessari per la diagnosi LND:

   1/2 3/5 5/10 >10

6. Presenza di SIB (comportamenti autolesionistici):

   SI NO (se NO, FINE TEST)

7. Età esordio SIB (in anni):

8. Tipologia del traumatismo:

   Labbra Lingua Dita Altro

9. E' stato utilizzato un dispositivo orale:

   SI NO (se NO, vai alla domanda 21)

10. Età di inizio uso dispositivo orale (in anni):

11. Numero di dispositivi orali utilizzati:

12. Tipologie diverse di dispositivi orali

SI NO

13. La famiglia ha riscontrato problemi estetici o sociali a causa del dispositivo orale?:

SI NO

14. Il paziente ha riferito problemi estetici o sociali a causa del dispositivo orale?:

SI NO

15. Ci sono state difficoltà nella gestione (inserimento, pulizia, tolleranza) del dispositivo orale?

SI NO

16. Sono insorti problemi articolari (mal di testa, dolori facciali, rumori articolari) a causa del dispositivo orale?:

SI NO

17. Sono insorti problemi parodontali (dolore alle gengive, abbondanti sanguinamenti gengivali) a causa del dispositivo orale?:

SI NO

18. Sono insorti problemi di tipo gastro/intestinale (dolori addominali, cattiva digestione, reflusso) a causa del dispositivo orale?:

SI NO

19. Il paziente si sente tranquillo o rassicurato o protetto nell'uso del dispositivo orale?

SI NO

20. La famiglia si sente più tranquilla quando il paziente usa il dispositivo orale?

SI NO

21. E' stata effettuata l'estrazione dei denti responsabili dei traumatismi?:

SI NO (se NO, FINE TEST)

22. Età in cui sono avvenute le estrazioni dentarie (anni):

23. Estrazioni dentarie in un’unica seduta?:

SI NO

24. La famiglia provava preoccupazione o timore prima delle estrazioni dentarie?:

SI NO

25. Il Paziente provava preoccupazione o timore prima delle estrazioni dentarie?:

SI NO

26. La famiglia riscontra problemi estetici o sociali a causa delle estrazioni dentarie?:

SI NO

27. Il Paziente riscontra problemi estetici o sociali a causa delle estrazioni dentarie?:

SI NO

28. Sono insorti problemi articolari (mal di testa, dolori facciali, rumori articolari) a causa delle estrazioni dentarie?:

SI NO

29. Sono insorti problemi di tipo gastro/intestinale (dolori addominali, cattiva digestione, reflusso) a causa delle estrazioni dentarie?:

SI NO

30. La famiglia si è sentita sollevata dopo le estrazioni dentarie?:

SI NO

31. Il paziente si è sentito sollevato dopo le estrazioni dentarie?

SI NO

32. Permane il SIB (comportamenti autolesionistici) dopo le estrazioni dentarie?

SI NO

Per ottemperare alla norma sul trattamento dei dati, si specifica che il Dott. Simone Buttiglieri, Odontoiatra, Dirigente Medico presso l'A.O. Ordine Mauriziano di Torino, reperibile all'indirizzo mail sbuttiglieri@mauriziano.it o al numero di telefono 011/5082389 è il titolare del trattamento dei dati personali.

Ai sensi dell'art. 13 del Regolamento UE 2016/679, La informiamo che:

- i dati di cui sopra sono raccolti per finalità di ricerca scientifica, sulla base del Suo consenso, liberamente prestato (mediante la compilazione del questionario) e revocabile in qualunque momento;

- non è obbligatorio fornire i dati e se non vorrà farlo non ci sarà alcuna conseguenza;

- i Suoi dati non verranno condivisi con nessun soggetto con finalità commerciali;

- le risposte verranno trattate in forma anonima e aggregata dal titolare del trattamento dei dati e da altri colleghi;

- in ogni momento potrà chiedere al titolare l'accesso ai suoi dati, la rettifica nel caso in cui siano incompleti o inesatti, la cancellazione, la limitazione del trattamento, o di opporsi al loro trattamento comunicando la revoca del consenso.

Firma del familiare/care giver del Paziente LND

____
